# Long-range selective transport of anions and cations in graphene oxide membranes, causing selective crystallization on the macroscale†

**DOI:** 10.1039/d0na00807a

**Published:** 2020-12-08

**Authors:** Vanesa Quintano, Alessandro Kovtun, Fabio Biscarini, Fabiola Liscio, Andrea Liscio, Vincenzo Palermo

**Affiliations:** Consiglio Nazionale delle Ricerche, Institute for Organic Synthesis and Photoreactivity, (CNR-ISOF) Via Gobetti 101 I-40129 Bologna Italy vincenzo.palermo@isof.cnr.it; Dipartimento di Scienze della Vita Via Giuseppe Campi 103 I-41125 Modena Italy; Consiglio Nazionale delle Ricerche, Istituto per la Microelettronica e Microsistemi, (CNR-IMM) – Sezione di Bologna Via Gobetti 101 I-40129 Bologna Italy; Consiglio Nazionale delle Ricerche, Istituto per la Microelettronica e Microsistemi, (CNR-IMM) – Sezione di Roma Via del fosso del cavaliere 100 I-00133 Roma Italy; Chalmers University of Technology, Department of Industrial and Materials Science Hörsalvägen 7 S-41296 Gothenburg Sweden

## Abstract

Monoatomic nanosheets can form 2-dimensional channels with tunable chemical properties, for ion storage and filtering applications. Here, we demonstrate transport of K^+^, Na^+^, and Li^+^ cations and F^−^ and Cl^−^ anions on the centimeter scale in graphene oxide membranes (GOMs), triggered by an electric bias. Besides ion transport, the GOM channels foster also the aggregation of the selected ions in salt crystals, whose composition is not the same as that of the pristine salt present in solution, highlighting the difference between the chemical environment in the 2D channels and in bulk solutions.

## Abstract 2

Ions can travel long distances within graphene-oxide membranes, and aggregate in microcrystals in between the sheets.

## Graphical abstract

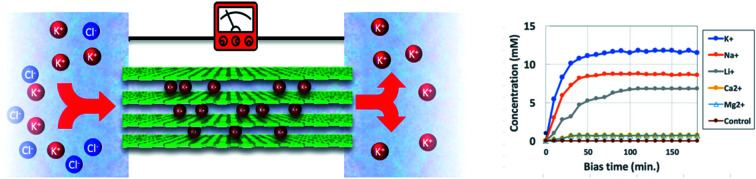

Synthetic nanofluidic devices are intensively studied with the final goal of achieving efficient ion sieving (for example for water desalinization), ion storage (for battery applications) or to mimic biological cellular membranes.^1^ Materials composed of stacked 2-dimensional nanosheets such as graphene can be used to produce large amounts of membranes featuring 2-dimensional channels precisely controlled at the nanometer scale and aligned parallel to the main membrane plane. In particular, graphene oxide (GO) can be easily processed in water as nanosheets having one-atom thickness and a lateral size of microns, then assembled in centimetre-size membranes where water can easily penetrate. The channel size can be easily tuned by tuning the oxidation grade of graphene,^2^ or using suitable spacers.^3–6^

Such graphene oxide membranes (GOMs) feature facile and scalable fabrication, high flux,^7^ efficient chemical modification, and tunable channel size. GOMs are intensively studied due to their great potential in scientific and practical industrial domains such as water treatment,^8–11^ desalination,^12^ and ion separation.^13,14^

Differently from what happens in bulk solutions, nanofluidic transport is largely governed by the surface properties of channel walls, particularly in low concentration electrolyte solutions.^15^ In sufficiently narrow nanochannels, the transporting molecules can be sieved by size exclusion or molecular recognition effects.^16^ Membranes composed of GO with polymer spacers can transport selectively molecules with different kinetic diameters in a gas,^17^ or capture efficiently small molecules in solution.^18^

Direct sieving of small ions by simple size exclusion is instead more challenging, due to a typical GO–GO sheet spacing *d* ≈ 9 Å, which is larger than the diameters of hydrated ions of most common salts. J. Abraham *et al.*^19^ described how to control *d* by physical confinement, describing membranes with *d* from ∼9.8 Å to 6.4 Å, providing a sieve size smaller than the diameters of hydrated ions.

Besides size exclusion, the ion transport behaviour can be also governed by the surface properties of the reassembled 2D nanosheets; in GOMs, the lamellar fluidic channels are fully covered with an electrical double layer (EDL) associated with the surface charge, which can be used to foster ion transport.^20^ The EDL is at the origin of various electrokinetic phenomena and its thickness (a.k.a. Debye length) results in the electrostatic exclusion of co-ions and enrichment in counter-ions, which affects the permselectivity of nanostructures.^21^

Ion sieving is typically achieved by applying high pressures, for example 10–100 bars in reverse osmosis (RO). However, nanofluidic ion transport in 2D nano-channels has also been demonstrated using electric fields.^22^ Such electrically driven ion transport can be achieved with simple, cheap setups and even used to create uni-directional ion pumps not only in GOMs^23^ but also in other layered materials such as V_2_O_5_.^24^

Most published studies on electric-field assisted ion transport focus on simple ions like K^+^ and H^+^; ion transport through GO-based membranes under the influence of an applied potential has been studied previously by Hong *et al.*,^25^ and further studies with MoS_2_ membranes have been conducted by Hirunpinyopas *et al.*^26,27^ The predominant transport mechanism has been attributed to the electrostatic attraction between the cations in solution and the negatively charged membrane channels, with atomic radii only affecting the transport above the critical channel width.^28^

In this work, we used electric fields to study the transport of different ions in GOMs between two vials connected through a mesoscopic GOM (Fig. 1a). In most published studies the transport is studied in the out-of-plane (OOP) direction, *i.e.* perpendicular to the main surface of the GO sheets, to achieve a high flux. Here, additionally, we studied ion transport in-plane (IP) as well, parallel to the GO sheets, which has some advantages *vs.* the standard OOP setup. Using the IP setup, the presence of small holes or nanometric defects in the membrane is less critical, because a single isolated hole will not cause a leak, differently from the OOP setup. Furthermore, the distance at which ions are transported can be truly macroscopic (centimeter scale) and easily controlled by tuning the GOM length. The ion flux can be tuned instead by varying the GOM width or its thickness (Fig. S11†). This versatility allows us to have source and drain solutions physically placed in different containers spaced apart, giving more experimental freedom; macroscopic GOMs can also be easily delaminated after use, to perform *ex situ* analysis of their composition. More importantly, the long-range transport on the centimeter scale allows us to maximize the time that the ions interact with the GO sheets within the nanochannels, thus exploiting at best any differential diffusion and migration mechanisms due to such interactions.

**Fig. 1:**
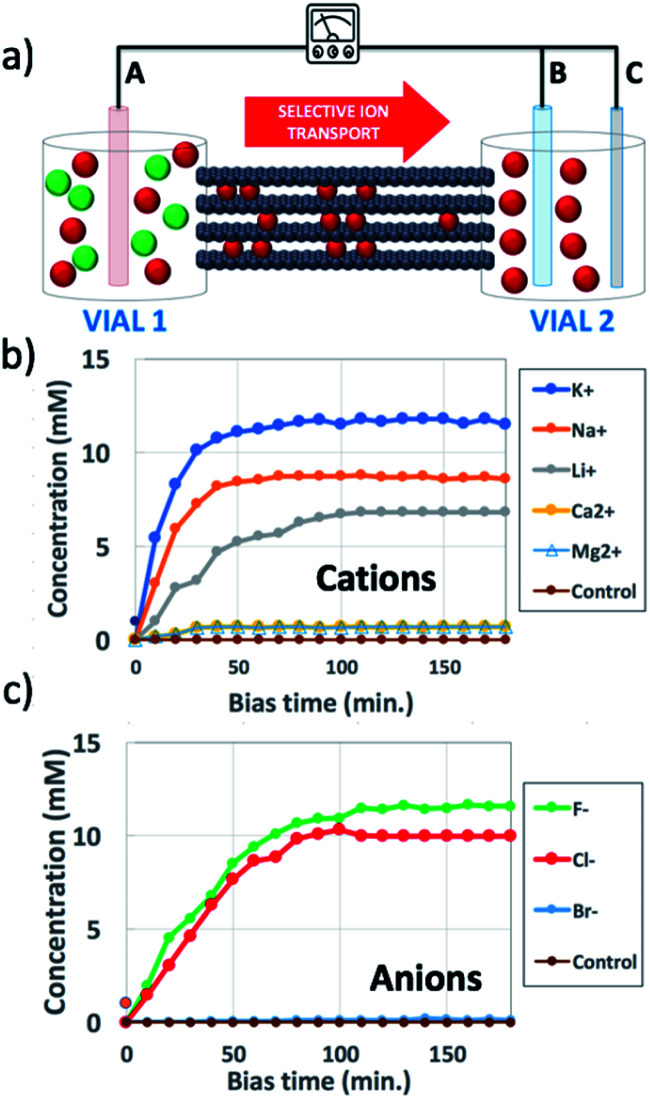
(a) Scheme of the set-up used: a steady bias *V*_AB_ is applied to B *vs.* reference electrode A for ion transport; a pulsed bias *V*_BC_ is applied to C *vs.* electrode B for ion detection. (b and c) Change in time of concentration in vial 2 of (b) cations, using *V*_AB_ = −0.5 V and (c) anions, using *V*_AB_ = 0.5 V. Concentration was estimated as described in Section 6 of the ESI.† Control was pure water. Counter-ions were always Cl^−^ for cations and K^+^ for anions.

Fig. 1a shows the typical IP setup. The initial concentration of salts was 100 mM in vial 1 and 0 mM in vial 2. The ion transport took place by applying a low electric bias (0.5 V) between two electrodes A and B, placed in vials 1 and 2 respectively, under static conditions of water flow. Under these conditions, ion transport due to osmotic pressure is not significant, as confirmed from control experiments with zero electric bias. The ion concentration in vial 2 due to ion migration was monitored using a pulsed electrochemical technique, applying a probe potential between B and a third reference electrode C, as detailed in the ESI (Fig. S6 and S7†). This method allowed a linear and reproducible measurement on the hour timescale without consuming or perturbing the ions in solution, as demonstrated by testing different solutions of variable known concentrations measured on different days (Fig. S8†). While applying the pulse between B and C, the bias between A and B was turned off, to avoid perturbation or cross-talk.

GOMs with high alignment of the sheets were produced by filtration from water solutions as detailed in Fig. S1 and Sections 1, 2 of the ESI.† The membrane thickness could be tuned by tuning the concentration of the initial GO solution (calibration curve available in Fig. S2†). Each GOM sample was coated on both sides with a hydrophobic film of polydimethylsiloxane (PDMS) in order to prevent water from passing on the external surfaces of the membrane (Fig. 2 and S3†). We studied transport of many small cations relevant for biological or energy storage applications (K^+^, Na^+^, Li^+^, Ca^2+^, and Mg^2+^) but also of the anions most commonly paired to such cations (F^−^, Cl^−^, and Br^−^). Thanks to the macroscopic size (3 cm length, 1 cm width) micrometric thickness and easy handling of the GOMs used, we could study and compare ion transport in both IP and OOP directions. After the ion transport experiments, the morphology of GOMs and the presence of the different ions were monitored using scanning electron microscopy (SEM), X-ray photoelectron spectroscopy (XPS), and X-ray diffraction (XRD) as detailed in Fig. S4 and Sections 3–5 of the ESI.†

**Fig. 2:**
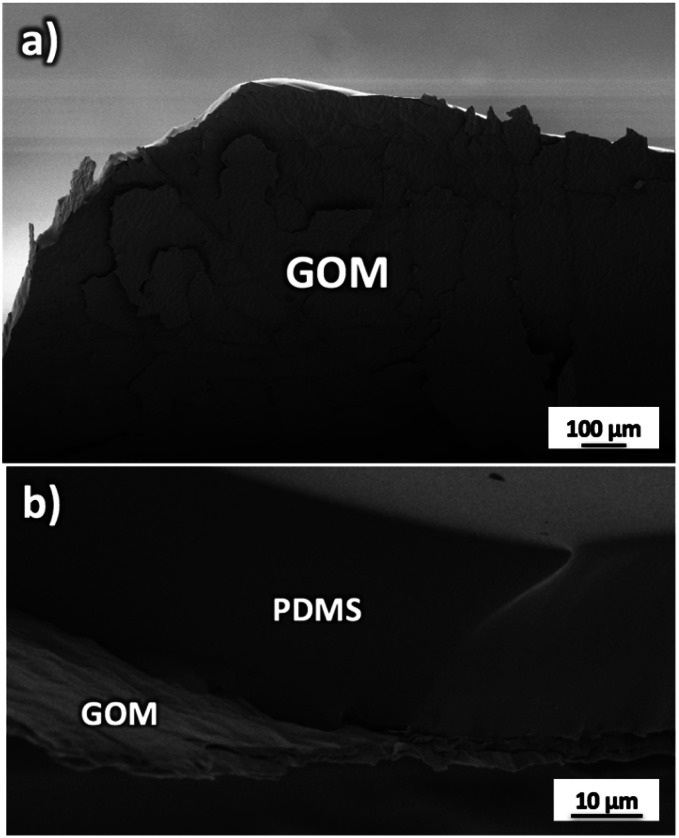
(a) SEM image of the GOM partially delaminated to show its structure. (b) Side view of the GOM attached to the supporting and protecting PDMS substrate.

Fig. 1b shows the concentration of different cations measured in vial 2 upon applying a bias *V*_AB_ = −0.5 V to electrode B for *t* = 180 min (in-plane configuration). K^+^, Na^+^, Li^+^, Ca^2+^, and Mg^2+^ were tested, having always Cl^−^ as the counter-ion. Fig. 1c shows the equivalent experiment done with anions, using *V*_AB_ = 0.5 V having K^+^ as the counter-ion. We can easily distinguish two stages: the ion concentration increased at first, then reached a plateau of stable concentration. While cations could be transported along the GOM thanks to the applied bias, no Cl^−^ ions reached vial 2, as confirmed also by *ex situ* chemical analysis (*i.e.* Cl^−^ titration with silver).

The behaviour observed is strikingly different from what was reported in previous studies in the OOP transport of GOMs, where permeability was clearly dependent on the hydrated diameter of ions,^11^ or on the kinetic diameter in case of gases.^17^ In the results obtained here, neither the height of the concentration plateau *C*_max_ nor the time needed to reach it seems to depend on ion size, hydrated or not. *C*_max_ showed some slight correlation with the hydrated radius and Stokes radius of the ions transported, with larger ions being slower in passing by; however, the linear correlation was not perfect, and there was an important exception for bromine (see Fig. 3 and S15 in the ESI,† and further discussion below).

**Fig. 3:**
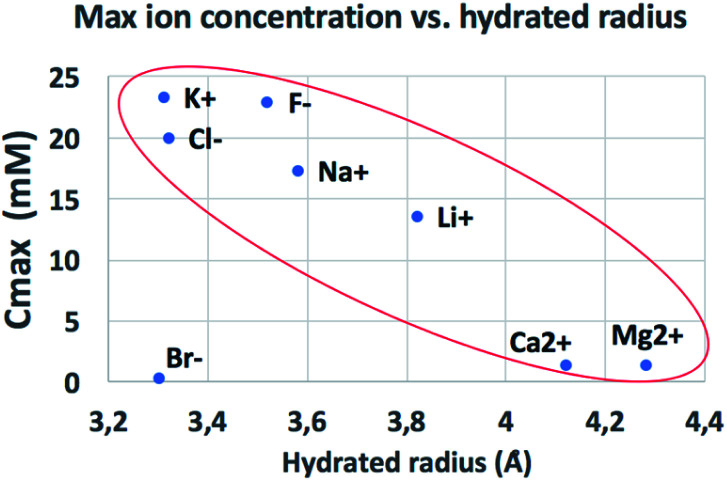
Comparison of the amount of ions transported in each experiment *vs.* ionic radius (see also Fig. S16† for correlation with other ionic properties).

Thus, additional mechanisms besides simple size exclusion should be present in the material.

We performed several blank experiments with (i) pure water and no salts, to ensure that the measured ions did not come from residuals present inside the GOM, (ii) with the same salts but using a non-selective microporous membrane (cellulose paper) between vials 1 and 2, which allowed fast diffusion of both cations and anions and (iii) with the same salts but using *V*_AB_ = 0. These control experiments confirmed that the transport was truly taking place along the GOM nanochannels and not along cracks or defects, and that an electric bias was necessary for ions to travel through the GOM.

Because the ion movement was caused by the presence of an external voltage gradient through the electrophoretic effect, the diffusion mechanism could thus be neglected;^29^ this could be expected, because ion diffusivity in 2D channels was much smaller than in bulk solutions (*D*_GOM_ ≪ *D*_bulk_).^30^

The presence of a concentration plateau was dependent only on the ion type used, and independent of the initial concentration. Tests performed with higher initial concentrations in vial 1 (up to 1 M) yielded the same concentration plateau in vial 2, different from what happens, for example, in classic diffusion processes.

The thickness of the GOM could be easily tuned during fabrication from 1.5 μm to 5.1 μm, tuning the amount of ions passing through the GOM (Fig. S11 in the ESI†). The time needed to reach the concentration plateau was always <40 min, regardless of the GOM thickness; though thicker GOMs gave a higher initial flux, allowing it to reach a higher final concentration in vial 2. These results suggest that the GOM acted as a series of channels in parallel, which can transport selectively cations or anions, where the channel length corresponds roughly to the GOM length. Eventually, the ion transport in the GOM channels stopped, yielding the concentration plateau observed in all samples.

The ion concentration dynamics is typically described by the Poisson–Nernst–Planck (PNP) equation.^31^ This equation describes the flux of ions under the influence of both an ionic concentration gradient ∇*c* and an electric field 
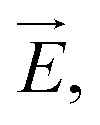
 also taking into account the electrostatic force between ions, as detailed in Section 7 of the ESI.†

We obtained a phenomenological description of the ion transport process by fitting all the concentration *vs.* time curves (Fig. 1b and c). All ions showing a significant transport in the GOM could be fitted with a stretched exponential (Fig. S9†). Stretched exponential behaviour has been observed in a wide range of chemo-physical phenomena; typically, this behaviour is used as a phenomenological description of relaxation in disordered systems and in the presence of parallel sequences of events, confirming the suggested model of GOMs as a set of multiple diffusion channels. However, this fit should be considered just qualitative, and cannot be correlated to a standard drift-diffusion mechanism (for example, the above mentioned PNP), because the concentration of the source in vial 1 changed with time (Fig. S12†) and the total amount of ions in the system composed of vial 1 and vial 2 was not constant due to ions crystallizing in the GOM, as detailed below.

We performed experiments measuring ion concentration in vials 1 and 2 at the same time, varying the orientation of the GOM as well, with the ion flux proceeding either in plane (IP, Fig. 4a) or out of plane (OOP, Fig. 4b, see also Fig. S12†) with respect to the membrane; photographs of the real setups used are available in Fig. S5 and S10 in the ESI.† In the IP setup, the ions had to travel the entire length of the membrane (3 cm). In the OOP setup, the thickness of the membrane separating vial 1 from vial 2 was only 1.5 μm; the effective tortuous path the ions had to travel was much longer than the real GOM thickness, due to the high aspect ratio of the GO sheets,^32^ but limited anyhow to few tens of microns (see ESI, Section 8† for details). Moreover, the GOM surface exposed to bulk solution and available for ion transport was much larger in the OOP than in the IP setup, providing a higher number of nano-channels able to transport ions in parallel.

**Fig. 4:**
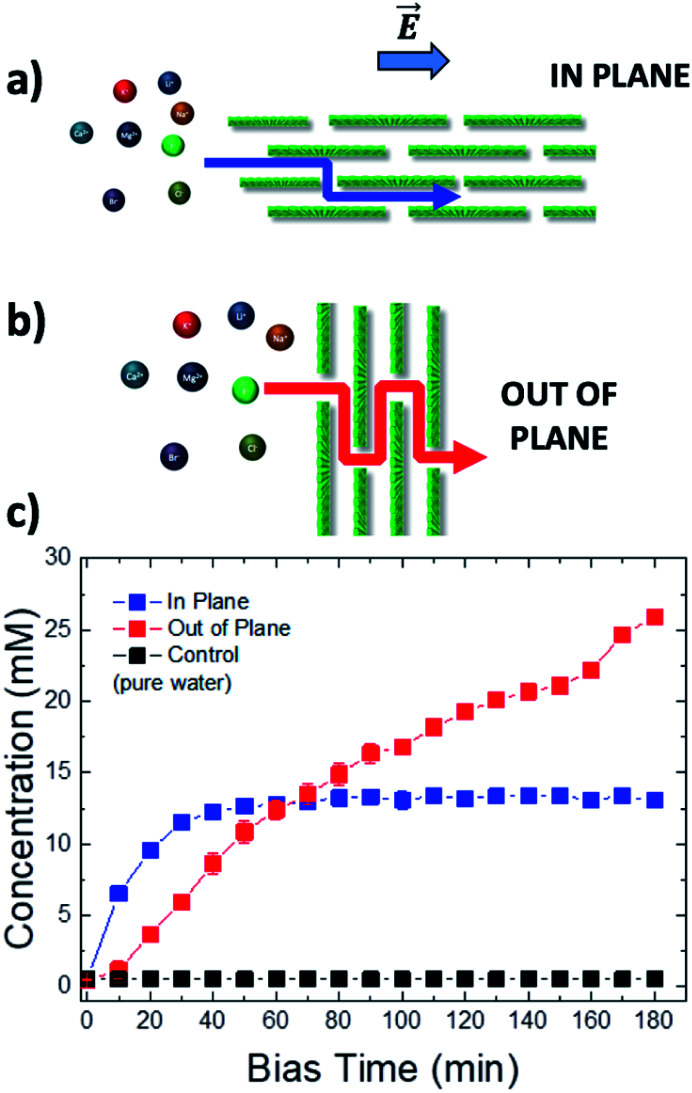
Schematic diagram of the tortuous ion path (red or blue line) in different experimental set-ups: (a) in plane (IP) and (b) out of plane (OOP). (c) Representative curves of the change in time of the concentration of K^+^ ions reaching vial 2 using the IP *vs.* OOP setup. Bias applied −0.5 V. The counter-ion was Cl^−^.

Due to the shorter path and a larger number of channels available, the transport could be very effective: in a control experiment using only cellulose as a separator in the OOP setup, the electric bias could force >90% of the ions to move from vial 1 to vial 2. Using instead a GOM in the OOP setup, we could again observe selective transport of ions, but with some significant difference with respect to the IP setup.

In the OOP setup the transport of ions increased linearly (red symbols in Fig. 4c), in good agreement with the standard electrophoretic mechanism, where the solution of the PNP equation is a linear function with slope = *v*_ion_/*L*_OOP_, and the ion velocity *v*_ion_ takes also into account the flow of the solvent (*i.e.* electro-osmosis, streaming current, *etc.*^20^).

This behaviour is much different from what was observed for the IP setup (blue symbols in Fig. 4c), where the time-dependence of the ion concentration seemed to stop, reaching a plateau at *ca.* 13 mM, being described by a stretched exponential function. Such behaviour could be caused by the presence of a “dissipative” term in the PNP equation which decreases the number of charges over time.

A possible cause of such behaviour could be the entrapment of charges in the GOM.

We decided thus to study not only the ion transport capability of the membrane, but also what remained in the channels after the ion transport, and we characterized *ex situ* the GOMs after using them for ion transport.

We investigated with X-ray diffraction (XRD) the presence of ions possibly intercalated inside the GOM (Fig. S13 and Table S1†). All spectra showed a peak due to the GO stacked sheets, but broader with respect to the one of the original GOMs, indicative of the partial disruption of the nanosheet stacking. A significant peak shift was observed in some cases, moving from the initial GO interlayer distance (*d* = 7.79 Å) to larger spacing (*d* > 9 Å), which could not be explained by ion intercalation between GO sheets. No clear correspondence was observed between the XRD spacing for a specific ion, its ionic radius and the ability of the GOM to transport such ions. In some cases, such as Na^+^, K^+^ and Cl^−^, a significant shift was observed; in others, such as Li^+^, *d* was comparable to the one of pristine GO. GOMs after passage of K^+^ or F^−^ gave also a more complex XRD signal and narrow Bragg peaks, indicating the presence of ion crystals. Thus, the obstruction of ion transport in GOMs could not be explained by the formation of classical GO-ion intercalation compounds.

Besides GO stacking, the presence of additional crystalline structures was observed in samples treated with different ions, in the form of XRD signals and diffraction peaks at higher *θ* angles, *i.e.* shorter periodicity (Fig. S13†) even if the intensity of the signal was not high enough to permit a certain assignment of the peaks.

SEM measurements allowed us to confirm eventually that the electro-driven transport of ions fostered ion crystallization inside the GOM, with the growth of well-defined crystals (Fig. 5; see also Fig. S14 and S15† for additional data). The crystals spanned all the GOMs and showed a preferential alignment along the direction of ion flux. The density of crystals observed was high for ions featuring a good transport in the GOM (Fig. 5), but very low for ions showing poor transport (see for example Ca^2+^, Fig. S14†). We did not observe the presence of any crystal or ions in either the pristine GO membrane or in the PDMS layers used to package the GOM, analyzed as the control.

**Fig. 5:**
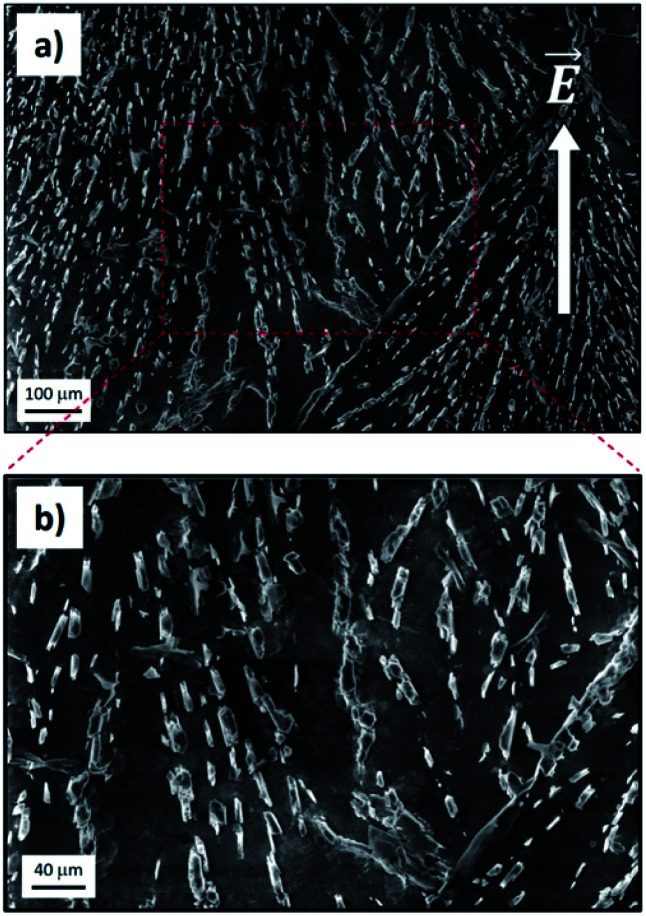
(a) Internal surface of the GOM as observed by SEM after K^+^ ion transport and subsequent peeling. The direction of macroscopic ion flux corresponding to the applied electric field is also shown (white arrow). (b) Magnification of (a).

We studied the chemical state of all ions using XPS, measuring the binding energy for photoelectron emission and the kinetic energy for Auger emission (Tables S2 and S3†). All elements transported in the GOM were present in ionic form, and the crystals were identified as salts. However, the salts formed inside the GOM did not always match the ones initially dissolved in vial 1 solution.

All cations were present without their initial anion (Cl^−^). Instead, anions could not penetrate alone into the negatively charged GOM, and were always observed in the presence of traces of their partner cations (K^+^).

XPS and EDS could determine only the chemical state of their atoms, not the exact formula of the compound present in the nanosheets; thus, different compounds could be present in the GOM for the specific ion. The XPS data indicated anyhow clearly that, while specific anions and cations could travel through GOMs, the transport mechanism was quite different.

Cations like K^+^, Na^+^ and Li^+^ could travel through the GOM likely interacting with the negatively charged hydroxyl and carboxyl groups present on the nanosheets' surface (GO has typically a zeta potential of −30 mV in bulk water at neutral pH).^33^

Divalent ions could not travel so effectively through GOMs because of their stronger interaction with GO surface groups, which make them also efficient cross-linker agents between stacked sheets; formation constant (stability constant) for Ca^2+^ and Mg^2+^ complexes with ligands possessing carboxylic moieties are higher than for Na^+^ and K^+^.^34^

Anions could be transported in GOMs, at least some of them (F^−^ and Cl^−^); however, different from cations, they were unable to travel on their own and were always found with their counter-cations as well. This could be explained assuming that, at pH = 7 used here, the surface of GO nanosheets was negatively charged, thus selective transport of anions would be hindered by the negatively charged groups present on the surface.^33^

The experimental results indicate that the transport process along GOMs can take place even on the centimeter scale, for both cations and anions; eventually, the transport stops, causing the plateau of concentration observed for all ions in Fig. 1b and c. This stop is attributed to ions accumulating within the channels at both the atomic scale (giving the changes in XRD observed) and at the mesoscopic scale, forming salt crystals (Fig. 5) which can grow to the micron scale, deforming and clogging the nano-channels of GOMs.

This evidence was confirmed by the anisotropic orientation of the crystals inside the GOM, indicating that the crystal grew during the directional flow of ions, roughly parallel to the applied electrical field, and not, for example, during the GOM drying and cutting operation performed for the analysis.

In the case of anions, which travelled together with their cations, the same salt present in solution was formed in the GOM. For cations, the situation was more complex; unfortunately, XPS measurements did not give clear results on the composition of the ions present in the GOM, which could be in the form of hydroxides or carbonates, nucleated by the –OH and –COOH groups already present on the nanosheet surface. Mesoscopic growth of hydroxides or carbonates cannot rely only on GO surface groups though, and can be explained by the presence of hydroxide (OH^−^) and bicarbonate (HCO_3_^−^) ions, coming from local variation of pH and carbonation of water. The crystals were distributed all along the GOM, another piece of mesoscopic evidence that the different ions can travel for many centimeters along the nano-channels, and be trapped inside it.

We compared the final concentration reached at the plateau (*C*_max_) observed for different properties of each ion, such as ionic radius, ionic crystal radius, hydrated and Stokes radius, electronegativity and ion charge/volume ratio (Fig. S16 and Table S4†). Besides the slight dependence on radius already mentioned above, no clear correlation could be obtained with any of these properties. Thus, the formation of specific salts in the GOM does not depend on a single property of the ion, but is instead caused by more complex interactions of the specific ion with the 2-dimensional chemical environment present in between the GO sheets.

The results obtained indicate that a great variety of ions can be easily transported in GOMs using an electric bias. The transport was observed with a low electric field applied (±0.5 V over a 3 cm distance). Besides simple transport, the ions showed also a complex chemistry inside the GOM, with significant phenomena of aggregations strongly dependent on the different ions present. The easy production of GOMs and their ability to intercalate and transport ions at the macroscopic scale make this approach highly suited to better unravel the chemistry inside 2D nano-confined systems, even if much remains to be understood of the chemistry and crystallization processes taking place in the constrained 2D nano-channels of GOMs.

## Conflicts of interest

There are no conflicts to declare.

## Supplementary Material

NA-003-D0NA00807A-s001
