# Supplementary material for: Long-range selective transport of anions and cations in graphene oxide membranes, causing selective crystallization on the macroscale
Source: Nanoscale Adv. 2020 Dec 8;3(2):353–8. doi: 10.1039/d0na00807a (PMC9418992; doi:10.1039/d0na00807a)
Supplement: NA-003-D0NA00807A-s001 [file NA-003-D0NA00807A-s001.pdf]

Electronic Supplementary Material (ESI) for Nanoscale Advances.
This journal is © The Royal Society of Chemistry 2020

Long-range selective transport of anions and cations in graphene oxide membranes, causing selective crystallization on the macroscale

Supporting information

CONTENTS:

1. **GO Preparation** 1
2. **GO Membrane Preparation** 2
3. **GOM structural characterization** 2
4. **X-Ray Diffraction (XRD)** 4
5. **GOM chemical characterization** 4
6. **Set-up for Electrochemical Measurements in plane** 5
7. **Ion concentration vs. time** 7
8. **Ion transport out of plane (OOP) of the GO membrane** 10
9. **Additional Figures** 11
10. **Additional Tables** 16
11. **References** 18

1. GO Preparation

Graphite was oxidized by a modified Hummers method to obtain water-soluble graphite oxide.[1] Briefly, graphite (100 mg) and sodium nitrate (100 mg) were added into 5 mL of fuming sulfuric acid (97-98%). Then, solid potassium permanganate (1 g) was slowly added to form the green manganese heptoxide (Mn_2O_7). The mixture was stirred for 3 days, diluted with sulfuric acid (5%; warning: this has to be done with extreme caution; the process is highly exothermic) and heated for 3 h to about 100-120 ◦C. After slow addition of 1 mL of hydrogen peroxide (30%), the resulting solid was collected by centrifugation and washed five times with sulfuric acid (3%) and hydrogen peroxide (3%), two times with hydrochloric acid (3.7%) and two times with water. Ions remaining in the solution were removed by dialysis against ultrapure water (3 days; water was changed every day). The water was removed by lyophilization to yield ~50 mg of water-soluble graphite oxide in the form of a light brown powder. The purity was checked by elemental analysis. The amount of

sulfur, nitrogen and chloride was under wt 0.1% each. Solutions of exfoliated GO were obtained by dissolving small amounts of graphite oxide in water (e.g. 0.5 mg/mL).

2. GO Membrane Preparation

The aqueous suspension of GO was prepared by dispersing GO flakes in distilled water using bath sonication for 4 hours. The resulted suspension was filtered onto an anopore inorganic membrane composed of aluminum oxide by vacuum filtration method (0.02 µm, diameter 47 mm, purchased from Whatman). The membranes were fished and cut into rectangular strips of dimension of 3×1 cm. The strips were then covered with a 500 µm PDMS slide both sides by an *in situ* cross-linking using a Film Applicator and Drying Time Recorder Coatmaster 510.

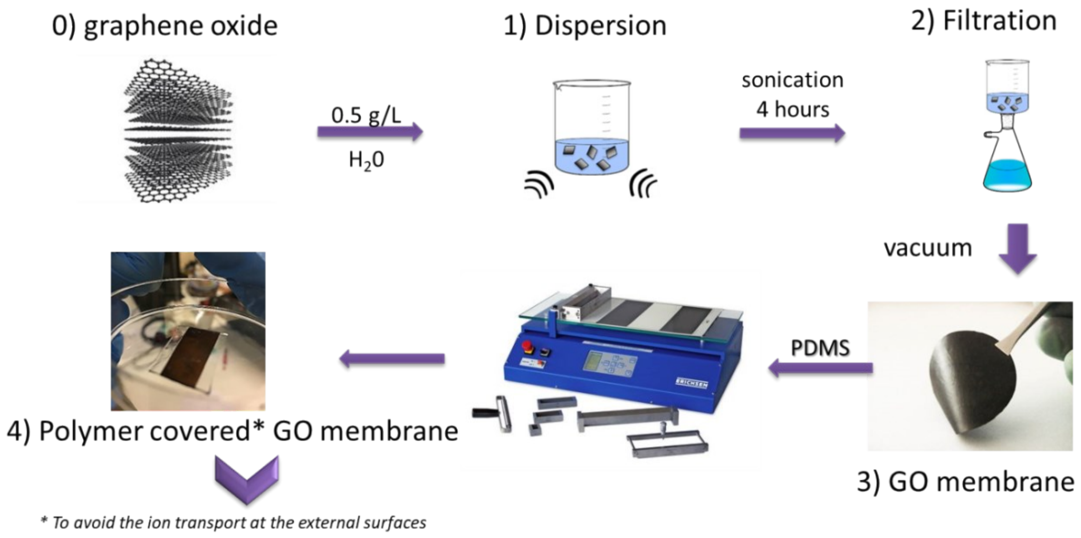

Figure S1. Scheme showing membrane fabrication.

PDMS was mixed in a 1:10 ratio in weight of Sylgard 184 Silicone Elastomer Curing Agent and PDMS monomers. The mixed ingredients were placed in a vacuum chamber for degassing under vacuum pressure for 30 minutes.

PDMS was used for preventing the transport of ions on the surface of the membrane and the disintegration of the GOM in water. PDMS is a polymer widely used for the fabrication and prototyping of microfluidic chips. Apart from microfluidics, it is used as a food additive (E900), in shampoos, and as an anti-foaming agent in beverages or in lubricating oils. It is transparent at optical frequencies (240 – 1100 nM), it has a low autofluorescence, it is considered as bio-compatible (with some restrictions), and it is deformable, which allows the integration of microfluidic valves using the deformation of PDMS micro-channels. It is easy to mold, because, even when mixed with the cross-linking agent, it remains liquid at room temperature for many hours.

3. GOM structural characterization

The thickness of the membranes could be tuned by varying the GO concentration in water during preparation. The measurement of the thickness was performed by SEM imaging of the membrane

cross-section. It showed a linear dependence on GO concentration in solution (fig. S2).

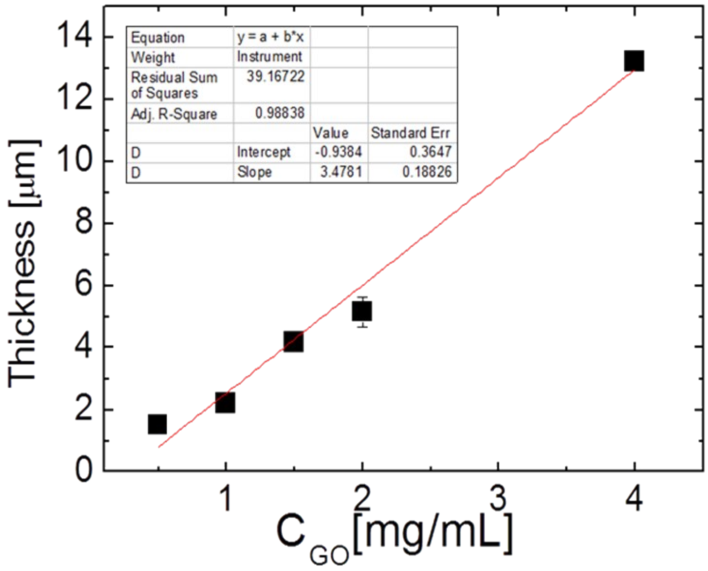

Figure S2. Membrane thickness vs GO concentration. The red line is the calibration curve calculated by the fit.

The uniformity of the film was confirmed by SEM measurements performed on the membrane after coating (fig. S3). In particular, a flat morphology was observed, with clearly visible flakes and stacked layers typical of 2-dimensional materials (fig. S3).

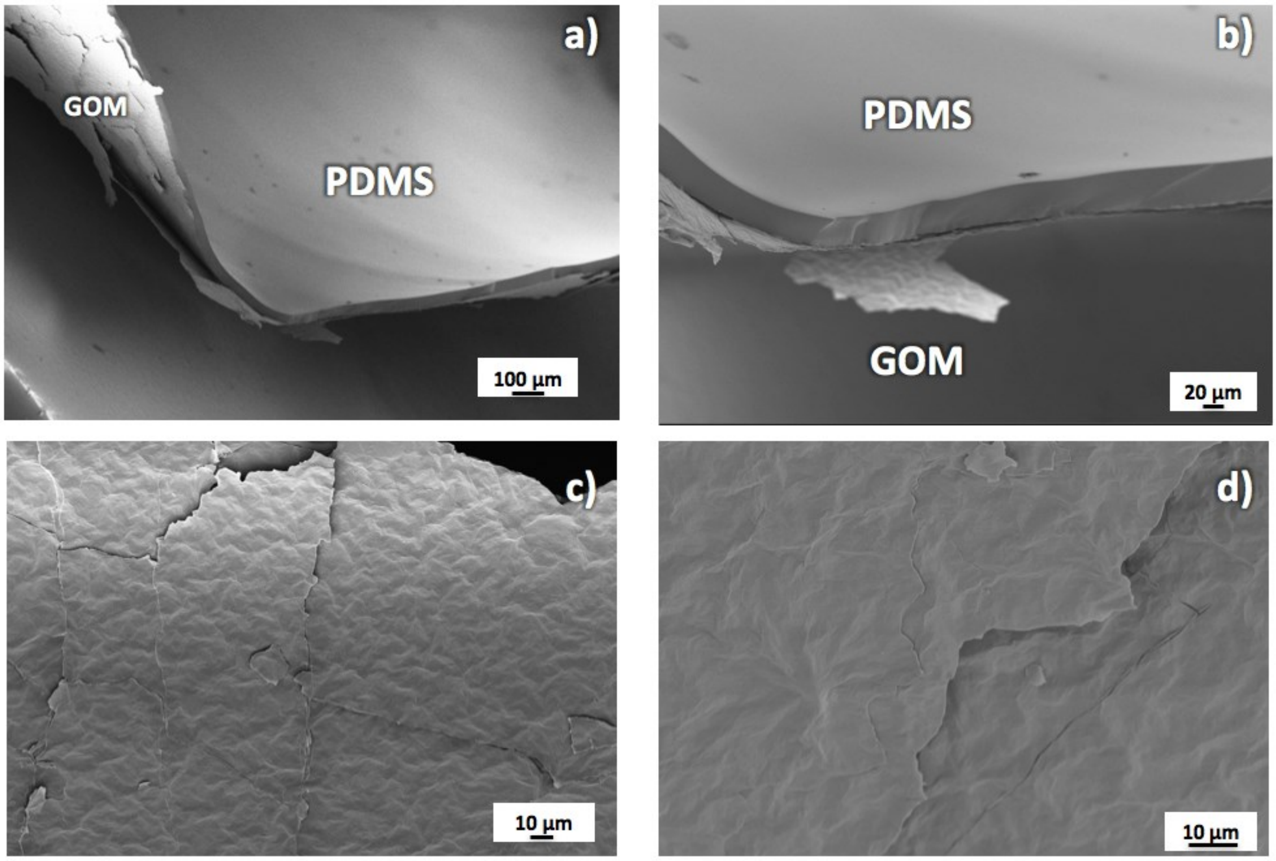

Figure S3. Additional SEM images of GOM partially delaminated to show their flexible structure, attached to

the supporting and protecting PDMS substrate.

4. X-Ray Diffraction (XRD)

X-Ray Diffraction (XRD) measurements were performed in specular geometry using a SmartLab-Rigaku diffractometer equipped with a rotating anode (Cu λ_α = 1.5405 Å), followed by a parabolic mirror to collimate the incident beam, and a series of variable slits (placed before and after the sample position) to reach an acceptance of 0.09°. Sample were prepared by mechanic peeling of the upper PDMS layers of PDMS/GO/PDMS membrane.

5. GOM chemical characterization

The chemical analysis of GO was performed using XPS. Sample were prepared by mechanic peeling of the upper PDMS layers of PDMS/GO/PDMS membrane after 2 hours of applied bias; pristine GO and pristine membrane on PDMS were also measured for comparison. Base pressure in the analysis chamber during analysis was 5 $\cdot 10^{-10}$ mbar. Data analysis and fitting were performed with CasaXPS software, after Shirley background subtraction.
Peak positions of the non-equivalent carbon species, based on literature data, were: aromatic carbon (C=C sp,2 284.4 eV), aliphatic carbon (C-C sp^3 , 285.0 eV), hydroxyl (C-OH, 285.7 eV), epoxy (C-O-C, 286.7 eV), carbonyl (C=O, 288.0 eV) and carboxyl (O-C=O, 289.1 eV).[2] All line-shape were fitted with Voigts function, the only exception was the asymmetric pseudo-voigt used for sp^2 aromatic carbon. All the doublets were fitted by a doublet with fixed spin orbit split of for Si 2p (0.63 eV), Ca 2p (3.60 eV), K 2p (2.77 eV), Cl 2p (1.6 eV) and Br 3p (1.05 eV) and a fixed area ration between the two components.
The pristine GO was mainly composed by Carbon and Oxygen, Sulphur and Nitrogen were present in small amounts. The Carbon to Oxygen ratio of GO is usually between 2 and 3. The C/O ratio was C/O=2.7±0.2, obtained by comparing carbon and oxygen 1s XPS peaks with a procedure specifically developed for GO characterization.[2]
The same measurement was performed at PDMS/GO/PDMS membrane, after the peeling of the upper PDMS layer: the C/O ratio seemed to increase, but extra amount of Carbon was due to the residual of PDMS. The formula of PDMS's monomeric unit is $-SiO(CH_3)_2-$, the amount of Si 2p signal was proportional to the amount of PDMS, which is about 13.9%. Silicon is present in all samples and can be associated to PDMS, moreover, a slight amount of Al (Al 2p 1.8%) was present to due to a residual of Alumina filter used for the preparation of GO membranes.
XPS spectra of all samples were obtained using a Phoibos 100 hemispherical energy analyser (Specs GmbH, Berlin, Germany) and Mg Ka radiation (ħω=1253.6 eV; power = 125 W) in constant analyser energy (CAE) mode, with analyser pass energy of 40 eV. The overall resolution of 1.5 eV was measured and analyser was calibrated using the Ag 3d 5/2 (368.3 eV) and Au 4f 7/2 (84.0 eV) signals from freshly Ar^+ sputtered samples. Charging effects were corrected by calibration of Binding Energy on C 1s (285.0 eV) for all spectra.

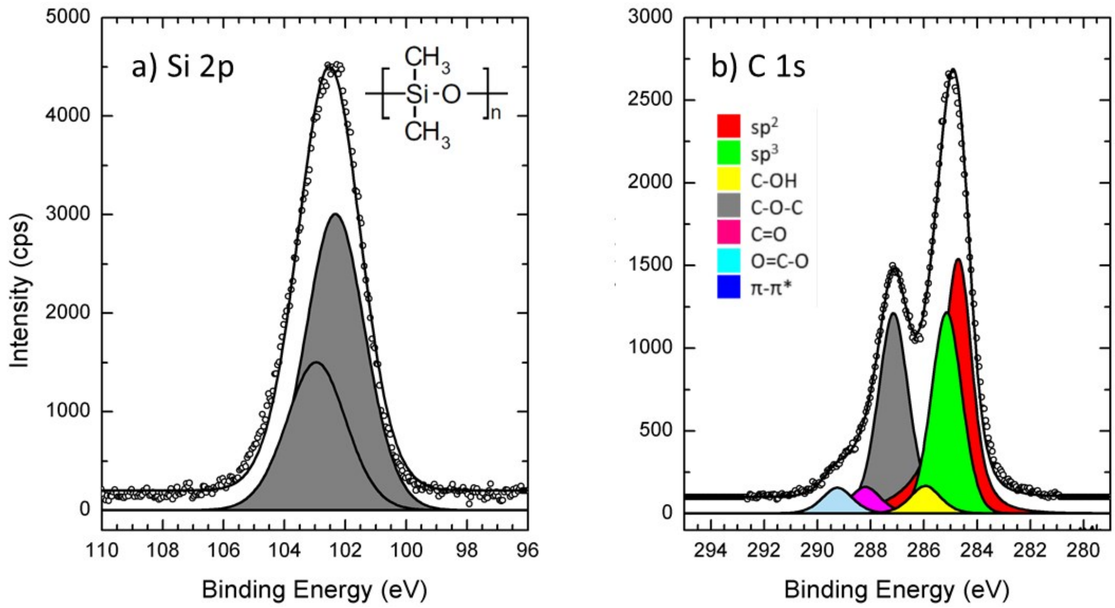

Figure S4. XPS spectra: A) Si 2p and b) C 1s of GO on PDMS.

6. *Set-up for Electrochemical Measurements in plane*

The experimental set-ups to measure ion transport in-plane (IP) along the GOM was composed by two vials (fig. S5). At the start of the experiment, vial 1 contained water/salt, vial 2 contained pure water. Vials 1 and 2 were then bridged by GOM, as shown in Fig. S5a. A DC electric potential was applied between electrodes A and B, placed in vials 1 and 2 respectively, to force ion transport across the GOM. Typically, electrode A was grounded. Cations could be attracted in vial 2 when electrode B was negatively polarized, anions when it was positively polarized. The typical biasing time needed to complete ion transport was 80 minutes, and the voltage applied was very low (+0.5V or -0.5V). To reduce noise and increase reproducibility, a compact setup composed of two separate compartments and a holder to fix the GOM was produced by 3D printing (fig. S5b).

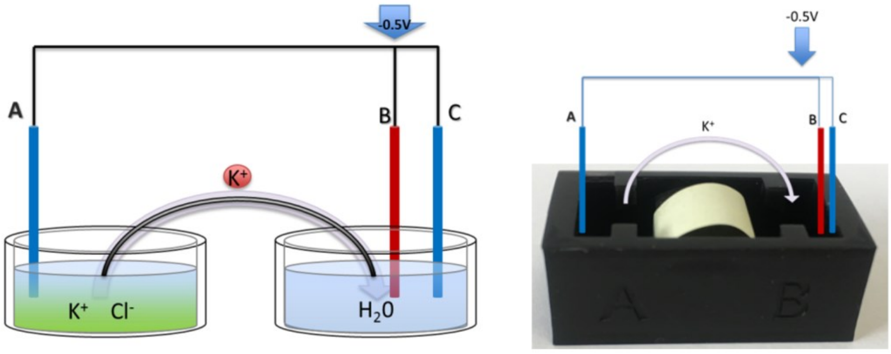

Figure S5. a) Set-up scheme. Bias applied between electrodes A and B to drive ion transport; pulsed bias applied between electrodes B and C for ion measurement. b) Scheme showing the real setup created by 3D printing.

A third electrode C was used to measure the concentration of ions transported in in vial 2.

Typically, ion concentration could be measured by measuring the ion conductivity of the solution present between B and C, by application of an additional bias. However, we did not use this conventional approach because the presence of an additional bias could perturb the ion transport between vials, creating artefacts.
Instead, we measured ion concentration with a pulse-method, which consists in applying a short voltage pulse between electrodes B and C.
A 1 second pulse was applied between B and C every 10 minutes. The electric pulse caused a transient current which was proportional to the concentration of ions in solution according to:

$$I = C\frac{dV}{dt}; \quad C = A\frac{\varepsilon\varepsilon_0}{\lambda_D}; \quad \lambda_D \propto \frac{1}{[M]} \qquad \text{S1}$$

where *I* is the measured current, *C* is the capacitance of the system, ε_0 is the value of the permittivity for air, ε is the permittivity of the dielectric medium and λ_D is the Debye length. The system was tested and calibrated by using salt solutions of precisely known concentration, showing a good linearity (fig. S6; Each concentration was measured 6 times to minimize the errors). The limit of detection of the method estimated in this way was 0.001 mM.

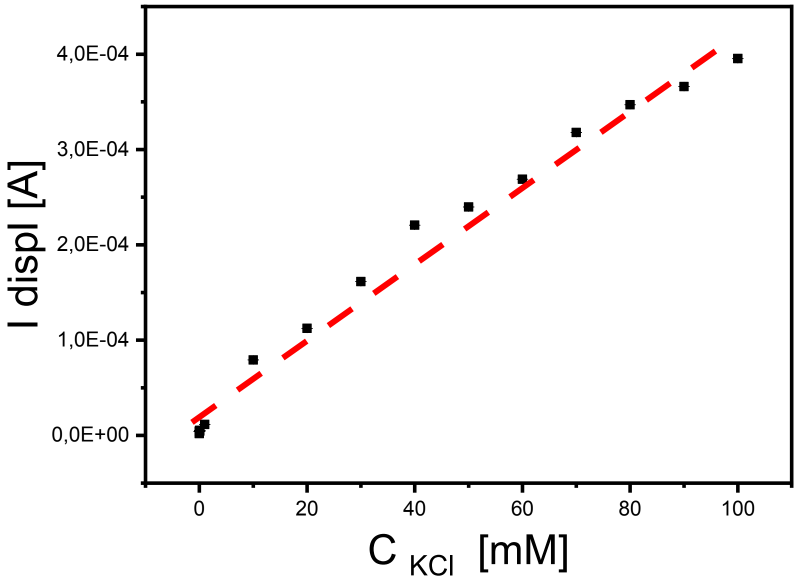

Figure S6. Correlation of the transient displacement current (I_{displ}) and the concentration of KCl.

Fig. S7 shows the typical bias applied and the resulting current transients observed at different times, during transport of K^+ ions across a GOM to vial 2. Fig. S7c shows the clear evolution in time of the current, which was able to measure the ion concentration without perturbing the main DC voltage applied between A and B.

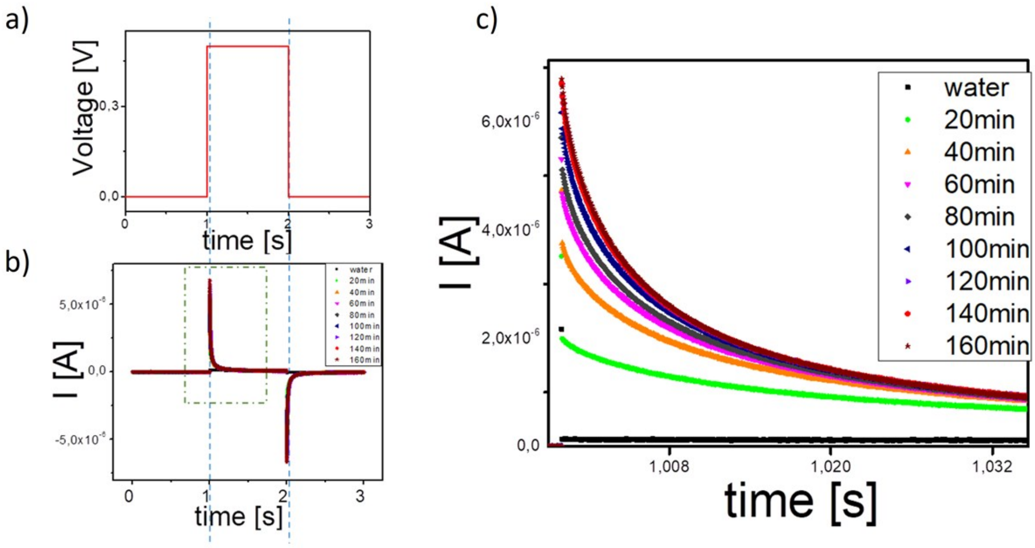

Figure S7. a) Applied pulse in BC. b) transient displacement current (I_{displ}) versus time. c) Zoom of b).

To ensure the reproducibility of the method, the same ion transport measurements were repeated in different days, with different ion solutions and different membranes. Fig. S8 compares two measurements taken in different days with different samples.

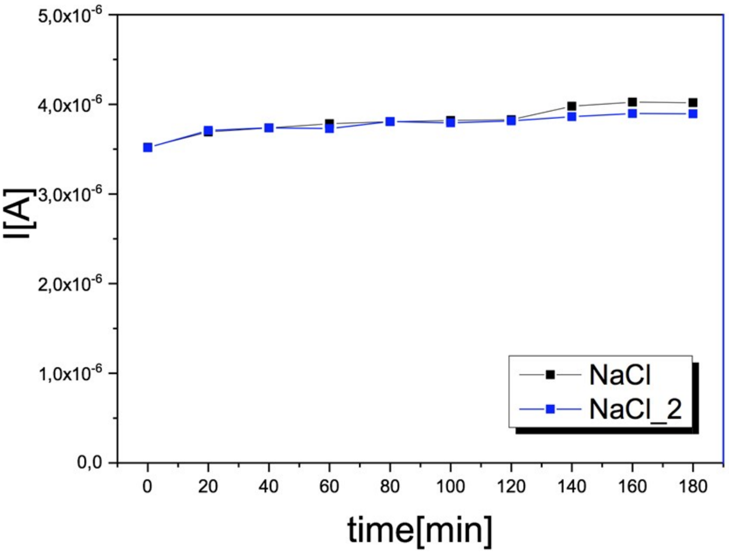

Figure S8. Max transient displacement current (I_{displ}) versus time measured in vial 2 for 2 different solutions of Na^+, with same concentration in vial 1 (0.1 M).

7. Ion concentration vs. time

The change in time of ions concentration *c(t)* is generally described by the Poisson-Nernst-Planck (PNP) equation.[3] This equation describes the flux of ions under the influence of both an ionic concentration gradient ∇c and an electric field $\vec{E}$, also taking into account the electrostatic force between ions. The equation in its general form can be written as:

$$\frac{\partial c}{\partial t} = - D \cdot \nabla\left[\nabla c + \frac{zF}{RT} c \cdot \vec{E}\right], \text{ where } \nabla\vec{E} = \rho + ze \cdot c \qquad \text{S2}$$

D and z are the bulk diffusivity and the valence values of the ion respectively, ρ the density of fixed charges. F is the Faraday constant, *e* the unit charge, *R* is the gas constant and T the temperature. When ions move in a channel, they can interact with the wall channel where ρ corresponds to the charge density due to electrical double layer (EDL).

The EDL plays a crucial role in nanofluidics governing different electrokinetic phenomena which can also show opposite contributions, such as electro-phoresis, electro-osmosis, streaming current, etc.[4]

For example, in the case of GOM the channel thickness is comparable to the EDL width (i.e. double of the Debye length). Thus, all the liquid in the channel is affected by electro-osmosis phenomenon (EO). This means that also in static condition (i.e. no pressure-driven case) the liquid in the channel flows in the opposite direction of ions with a velocity roughly proportional to the applied bias. In first approximation the effective ion mobility is the sum of two terms:

$$\mu_{eff} = \mu_0 - \mu_{EO} \qquad \text{S3}$$

The first term μ_0 is the intrinsic ion mobility calculated by the Einstein relation and depends on the ion; the second term depends also on the properties of the liquid and the channel walls. Using the Smoluchowski formula[5] we can write the equivalence: $\mu_{EO} = \frac{\varepsilon}{\eta} \cdot \zeta$, where ε the dielectric constant, η the viscosity and ζ the Zeta (electrostatic) potential.

Considering the values of bulk water ($\varepsilon = 6.94154*10^{-10}$ F/m, $\eta = 7.98*10^{-4}$ N·s/m^2)[6] and Zeta potential of GO = -35 mV, as measured by Dynamic Light Scattering;[7] we obtain a value of -3.04*10^{-8} m^2/s/V which is comparable to the intrinsic mobility of the ions studied in this work.

We performed a phenomenological description of the change in time of ion concentration. For all the ions, we found that a stretched exponential curve best reproduced the experimental trends:

$$C = C_{max} \cdot \left(1 - exp\left\{-\frac{t}{\tau}\right\}^{\alpha}\right) \qquad \text{S4}$$

where C_{max} is the final concentration, τ is a characteristics transition time and α is a parameter indicating the dispersion of drift velocities in the material. We could linearize all the data and use a least square linear fit with the simple transformations:

$$A = ln\left(\frac{C_{max}}{C_{max} - C}\right) = - ln\left(1 - \frac{C}{C_{max}}\right) = \left({}^{t}/_{\tau}\right)^{\alpha} \qquad \text{S5}$$

$$\ln(A) = \alpha \ln(t) - \alpha\, ln(\tau) \qquad \text{S6}$$

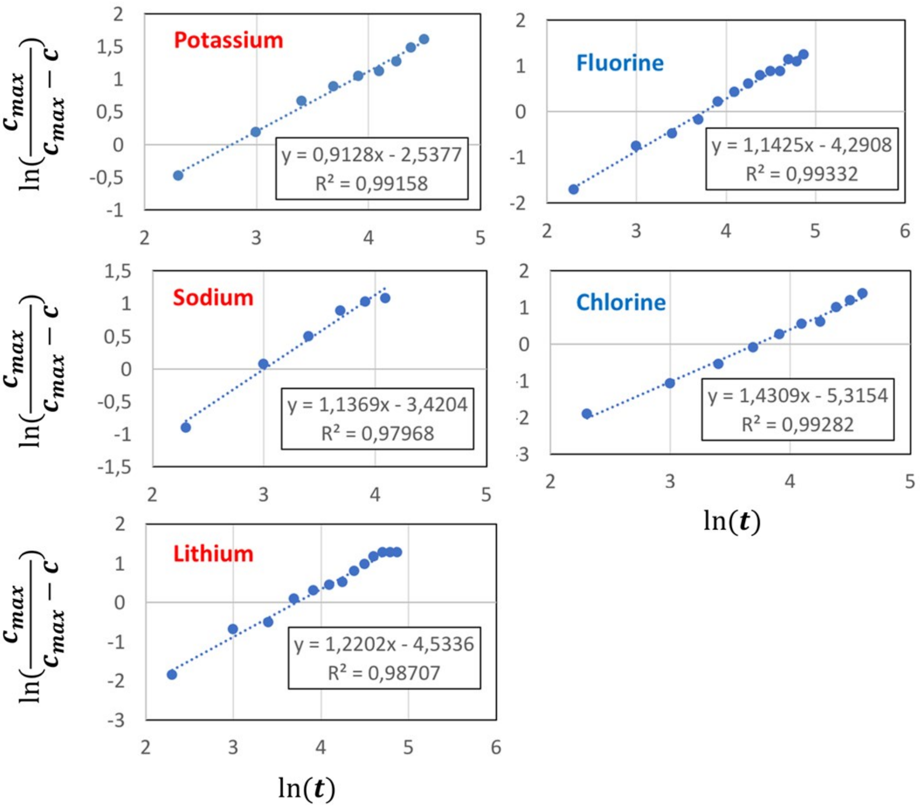

Figure S9. Log-log plot of parameter a versus time t measured during concentration increase in vial 2. The dotted lines indicate the linear fit. In each inset, the fitting parameters and the R^2 value calculated.

All ions showing a significant transport in the GOM could be fitted with this equation (fig. S9). Stretched exponential behaviour has been observed in a wide range of chemo-physical phenomena; typically, this behaviour is used as phenomenological description of relaxation in disordered systems and in presence of parallel sequence of events, confirming the suggested model of GOM as a set of multiple diffusion channels. However, this fit should be considered just qualitative, and cannot be correlated to a standard drift-diffusion mechanism, because the concentration of the source vial 1 changed with time and the total amount of ions in the system composed by vial 1 and vial 2 was not constant due to ions crystallizing in the GOM (see main text).

8. Ion transport out of plane (OOP) of the GO membrane

To measure ion transport across GOM, we connected two vials by a tailored membrane holder which is placed the membrane in vertical position between two vials 1 and 2. We used the same electrode setup used for in-plane measurements for OOP.

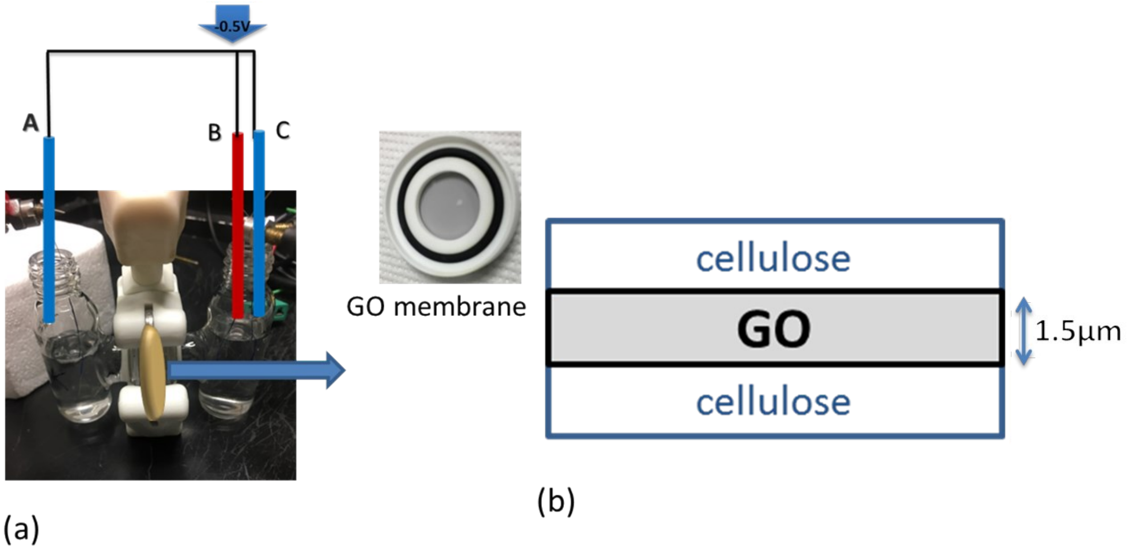

Figure S10. (a) Photograph showing GO out of plane device. (b) GO strip membrane covered with porous cellulose to prevent GO degradation.

To allow OOP transport but avoid any mechanical deformation or degradation of the GOM, the sample was not embedded in PDMS but in two macro-porous cellulose layers, perfectly permeable to water and all ions.

The effective length that ions have to travel in OOP setup is larger than the GOM thickness because, due to the 2D shape of the stacked GO sheets, the ions have to follow even in this case a tortuous path. Considering a GOM thickness $T=1.5\ \mu m$, an inter-sheet spacing $d\approx 1\ nm$ and an approximated lateral width of each sheet $w\approx 50\ nm$ [8] the estimated length of the tortuous path that ions had to diffuse to cross the membrane was $L \sim Tw/d$, in the few tens of microns scale, instead of the 3 cm used for IP experiments.

9. Additional Figures

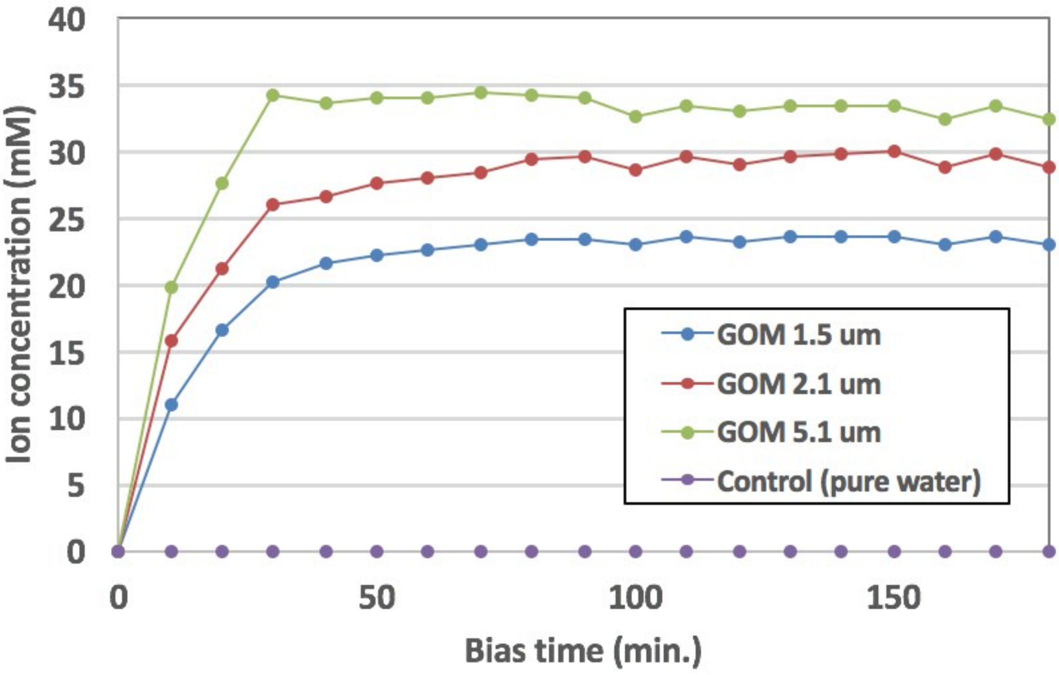

Figure S11. Change in time of concentration of K^+ ions diffusing from vial 1 to vial 2 in IP setup, using GOM of different thickness. Initial concentration in vial 1 C =100 mM). Bias applied -0.5 V, GOM thickness: 1.5±0.1 µm, 2.1±0.1 µm and 5.1±0.3 µm. A blank sample with bias applied on pure water is also reported for comparison.

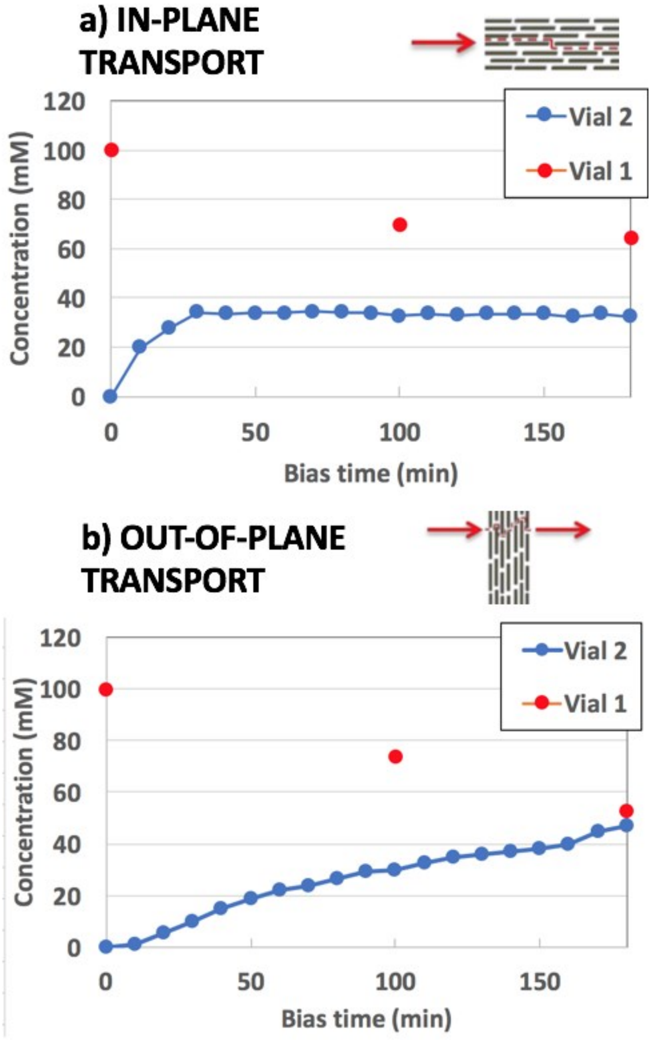

Figure S12. Ion concentration vs time of biasing in vial 1 (black), and vial 2 (red) in: a) In plane; b) Out of plane.

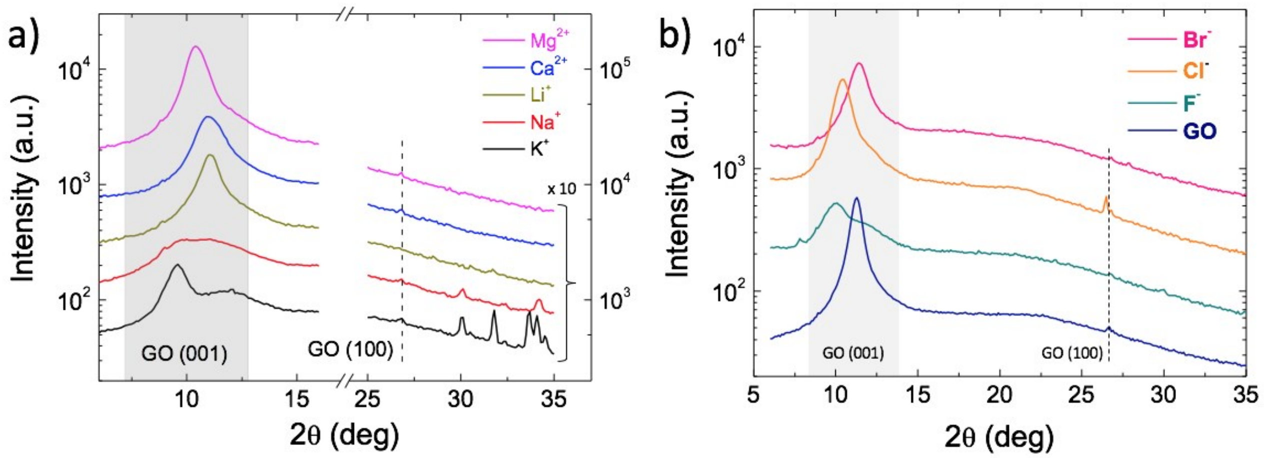

Figure S13. XRD spectra showing the change in the d-spacing of GO membranes in different salt solutions (gray-shaded area) and the presence of additional peaks of low intensity at higher angles, due to the crystals formed: a) cation (fixed anion = Cl^-), b) anion (fixed cation = K^+).

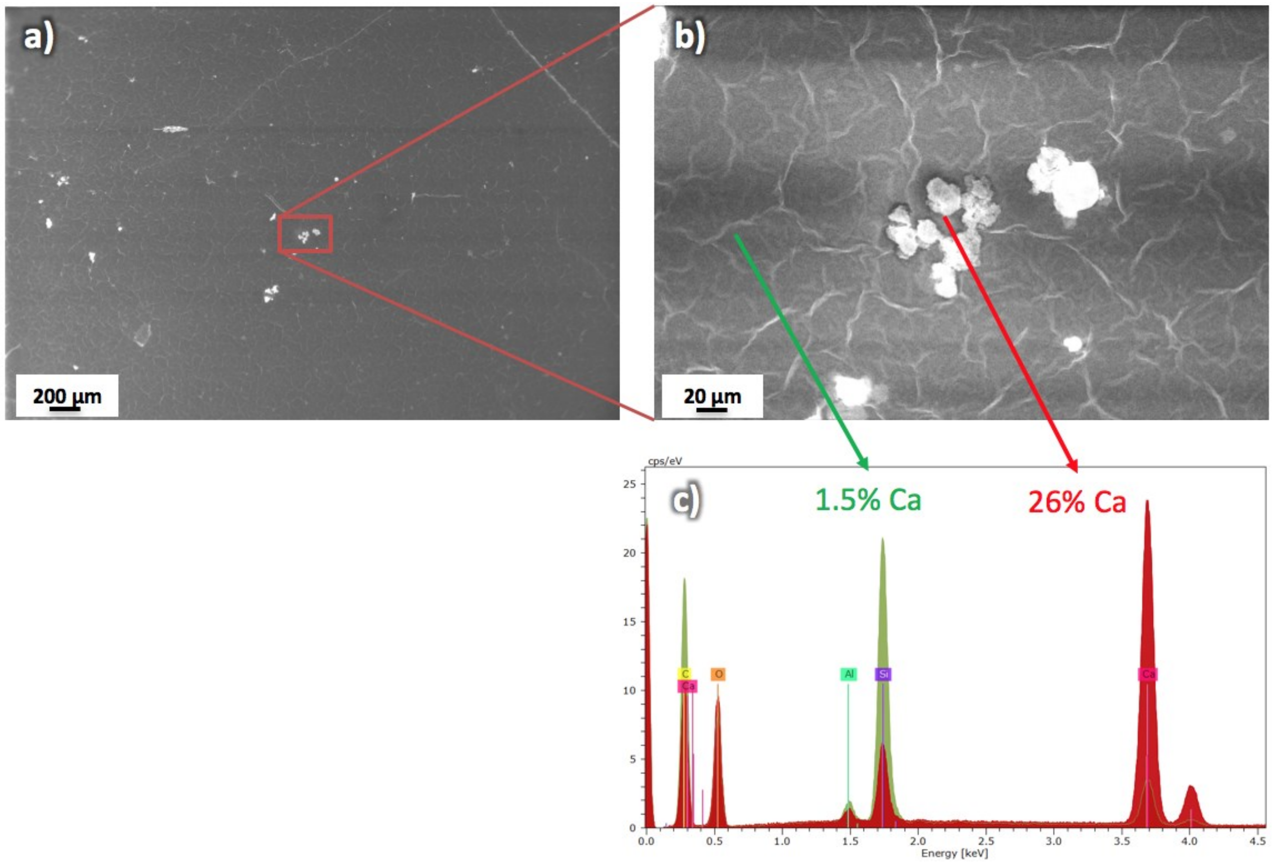

Figure S14. SEM images of the inner side of GOM after Ca^{2+} ion transport experiments and subsequent peeling, showing presence of sparse crystalline agglomerates. b) zoom-in of a). c) EDS analysis of different areas in the sample, showing that the observed crystals are mainly composed of Ca.

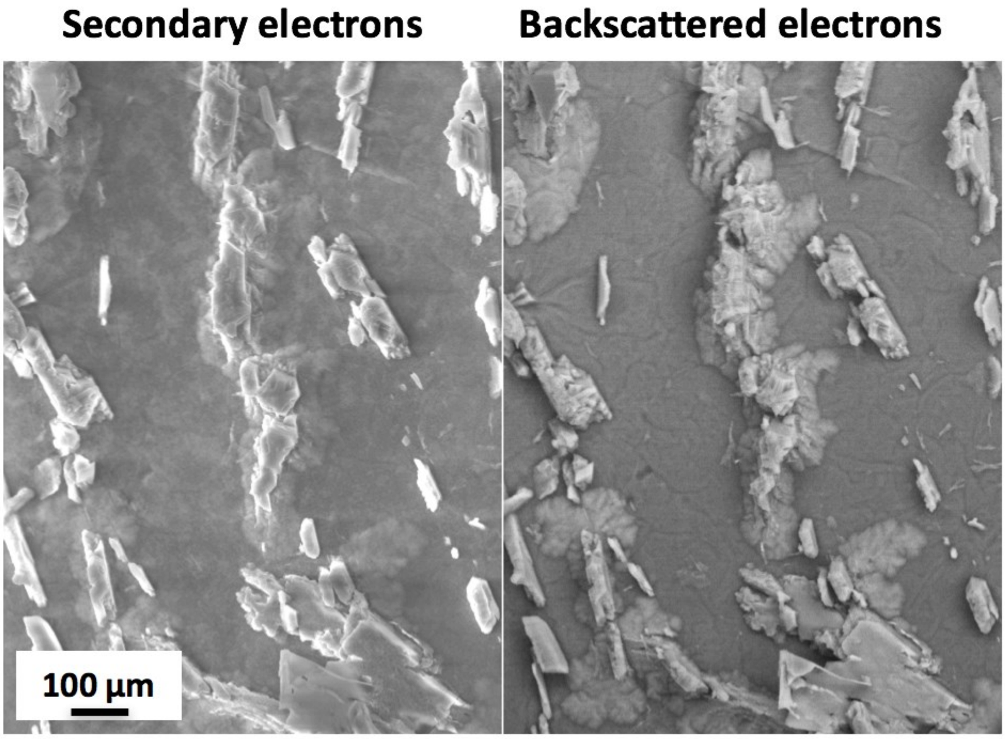

Figure S15. SEM images of the inner side of GOM after K^+ ion transport experiments and subsequent peeling, showing presence of sparse crystalline agglomerates. a) secondary electrons. B Back-scattered electrons, showing different contrast between the crystals and the GOM membrane.

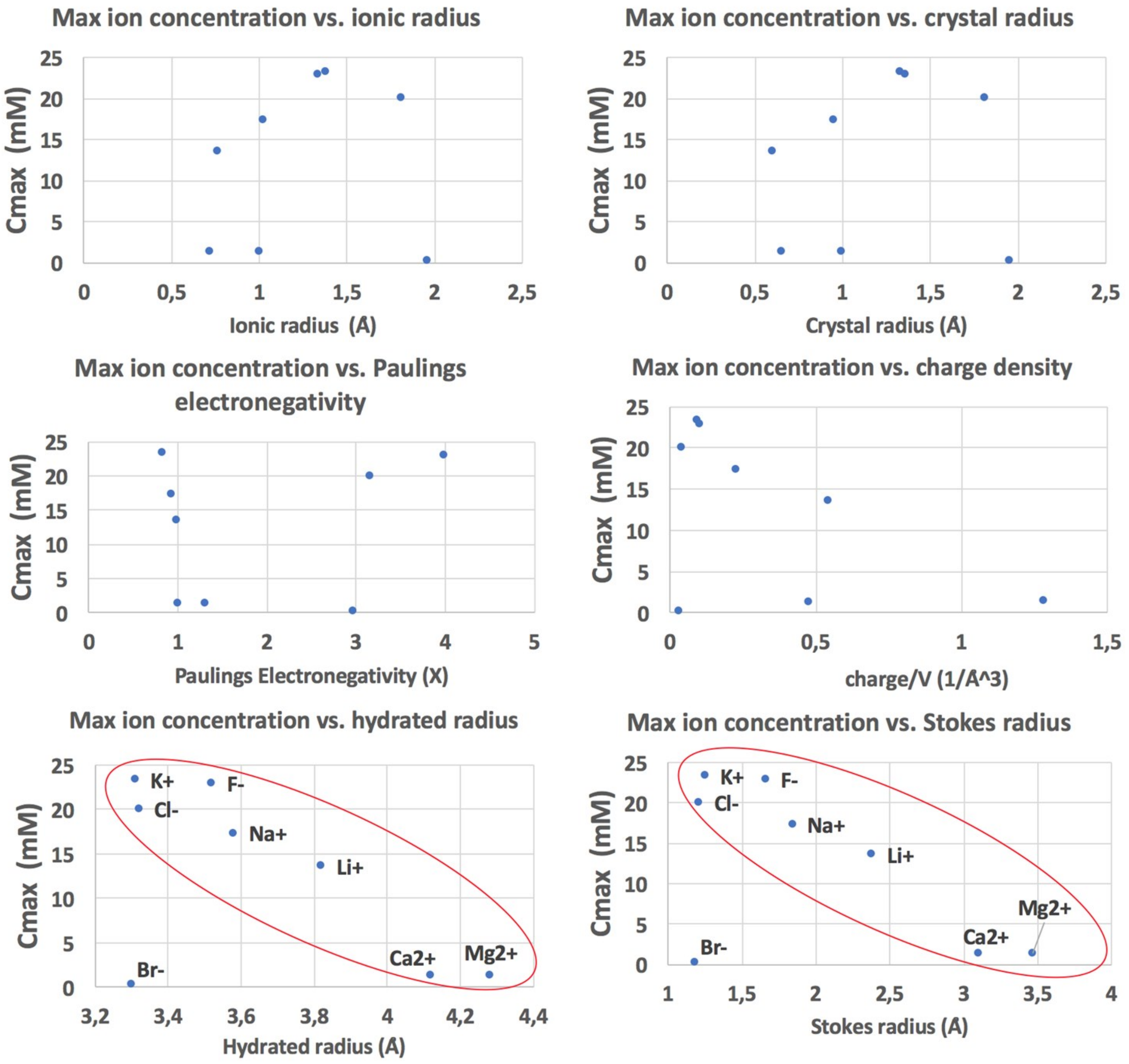

Figure S16. Comparison of the amount of ions transported in each experiment (measured as the final concentration of ions in vial 2) vs. specific parameters of the ions: ionic radius, ionic crystal radius, hydrated and Stokes radius, electronegativity and charge density (number of charges / ion volume). Values obtained from table S4 here below. The red circles are just a guide for the eye.

10. Additional Tables

Table S1. XRD spacing of GO membranes in different salt solutions: (A) cation (fixed anion = Cl^-), (B) anion (fixed cation = K^+).

Initial Salt	**Ion**	**XRD periodicity (Å) [error =0.01 Å]**	**Hydrated radius (Å)**
LiCl	Li^+	7.99	3.82
NaCl	Na^+	9.20 / 7.54	3.58
KCl	K^+	9.24 / 7.44	3.31
CaCl2	Ca^{2+}	7.99	4.12
MgCl2	Mg^{2+}	8.45	4.28
KF	F^-	8.85 / 7.48	3.52
KCl	Cl^-	8.48/ 7.29	3.32
KBr	Br^-	7.74	3.30

Table S2. Identification of cations present on GO membrane in PDMS after bias: characteristic transitions observed and reference values. KE=Kinetic Energy, BE=Binding Energy.

**Sample**	**Transitions**	**Peak position (eV)**	**Reference values (eV)**	**Chemical state**	**At. % Cation**
**GO**	C 1s	284.4 ± 0.1 (BE)	-	-	-
**Li^+**	Li 1s<br>C 1s	55.1 ± 0.1 (BE)<br>289.9 ± 0.1 (BE)	54.9 [10]<br>289.6	Li^+	22.9 ±0.8
**Na^+**	N 1s<br>Na KLL	1072.5 ± 0.1 (BE)<br>988.8 ± 0.1 (KE)	1071.5-<br>1072.5 [9]	Na^+	2.2 ±0.2
**K^+**	K 2p3/2	293.2 ± 0.1 (BE)	292-294 [9]	K^+	6.7 ±0.4
**Ca^{++}**	Ca 2p3/2	347.8 ± 0.1 (BE)	347.5-348 [9]	Ca^{++}	1.2 ±0.2
**Mg^{++}**	Mg 2p3/2<br>Mg KLL	51.2 ± 0.1 (BE)<br>947.0 ± 0.1 (KE)	51-51.5 [9]	Mg^{++}	1.4 ±0.2 *

Table S3. Identification of anions present on GO membrane in PDMS after bias: characteristic transitions observed and reference values.

Sample	Transitions	Peak position (eV)	Reference values (eV)	Chemical state	At. % Anion
F^-	F 1s<br>F AP<br>K 2p3/2	685.6±0.1 (BE)<br>1340.3±0.1(BE)<br>347-348 (BE)	1340.2 [11]	CaF_2<br>Or KF	16.8 ±0.6
Cl^-	CL 2p3/2<br>K 2p3/2	197.4±0.1 (BE)<br>292.6±0.1 (BE)	198.5 [9]	KCl	1.3 ±0.2
Br^-	Br 3d5/2<br>K 2p3/2	68.3±0.1 (BE)<br>293.2±0.1 (BE)	68.8 [9]<br>292.2	KBr	0.7 ±0.2

*Table S4. Parameters of the ions under study and the salt used in the experiments. * The parameter Calculated Effective Density (C/Å^3) is an estimated value of number of charge / hydrated volume.*[12, 13]

	Li+	Na+	K+	Ca2+	Mg2+	F-	Cl-	Br-
**Ionic radius (Å)**	0.76	1.02	1.38	1.00	0.72	1.33	1.81	1.96
**Crystal radius (Å)**	0.60	0.95	1.33	0.99	0.65	1.36	1.81	1.95
**Stokes radius (Å)**	2.38	1.84	1.25	3.10	3.47	1.66	1.21	1.18
**Hydrated radius (Å)**	3.82	3.58	3.31	4.12	4.28	3.52	3.32	3.30
**Paulings Electronegativity (X)**	0.98	0.93	0.82	1	1.31	3.98	3.16	2.96
**Hydrated volume (Å^3)**	233	1.92	152	293	328	183	153	151
**Effective density (×10^{-22} C/Å^3)**	6.86	8.34	10.5	5.47	4.88	-8.77	-10.5	-10.6
**Charge densities (C/mm^3)**	52	24	11	52	120	24	8	6
**C_{max} (mM)**	6,8 ± 0,3	8,6 ± 0,4	11,50 ± 0,04	0,702 ± 0,001	0,688 ± 0,002	11,58 ± 0,07	9,98 ± 0,11	11,58 ± 0,07

11. References

1. Hummers, W. S. & Offeman, R. E. Preparation of Graphitic Oxide. *J. Am. Chem. Soc.* **80**, 1339 (1958).
2. Kovtun, A. *et al.* Accurate chemical analysis of oxygenated graphene-based materials using X-ray photoelectron spectroscopy. *Carbon N. Y.* **143**, 268–275 (2019).
3. Bazant, M. Z., Thornton, K. & Ajdari, A. Diffuse-charge dynamics in electrochemical systems. *Phys. Rev. E - Stat. Physics, Plasmas, Fluids, Relat. Interdiscip. Top.* **70**, 24 (2004).
4. Bocquet, L. & Charlaix, E. Nanofluidics, from bulk to interfaces. *Chem. Soc. Rev.* **39**, 1073–1095 (2010).
5. Hunter, R. J. *Foundations of Colloid Science (2nd Edition)*. *Oxford University Press* (Oxford University Press, 2001).
6. Atkins, P. & Paula, J. De. Atkins' Physical chemistry 8th edition. *Chemistry* 430–468 (2009). doi:10.1021/ed056pA260.1
7. Li, D., Müller, M. B., Gilje, S., Kaner, R. B. & Wallace, G. G. Processable aqueous dispersions of graphene nanosheets. *Nat. Nanotechnol.* **3**, 101–105 (2008).
8. Erickson, K. *et al.* Determination of the local chemical structure of graphene oxide and reduced graphene oxide. *Adv. Mater.* **22**, 4467–4472 (2010).
9. Moulder, J., Stickle, W., Sobol, P. & Bomben, K. Handbook of X-ray Photoelectron Spectroscopy Perkin-Elmer Corp., Physical Electronics Division, Eden Prairie, Minnesota, USA, 1979. *Surf. Interface Anal.* (1981). doi:10.1002/sia.740030412
10. Contarini, S. & Rabalais, J. W. Ion bombardment-induced decomposition of Li and Ba sulfates and carbonates studied by X-ray photoelectron spectroscopy. *J. Electron Spectros. Relat. Phenomena* **35**, 191–201 (1985).
11. Briggs, D.Wanger, C. D., Riggs, W. M., Davis, L. E., Moulder, J. F., Muilenberg, G. E. Handbook of X-ray Photoelectron Spectroscopy: a reference book of standard data for use in x-ray photoelectron spectroscopy. *Perkin-Elmer Corporation* (1979).
12. Marcus, Y. A simple empirical model describing the thermodynamics of hydration of ions of widely varying charges, sizes, and shapes. *Biophysical Chemistry* **51**, 111–127 (1994).
13. Nightingale, E. R. Phenomenological theory of ion solvation. Effective radii of hydrated ions. *J. Phys. Chem.* **63**, 1381–1387 (1959).
